# An image analysis algorithm based on the hue saturation density transformation, an important tool for melanoma immunotherapy research

**DOI:** 10.1186/2051-1426-2-S3-P134

**Published:** 2014-11-06

**Authors:** Jeffrey A Dzubay, Alan Jerusalmi, Rajiv Jesudason, Johannes Zimmermann, Martin Baatz, Zipei Feng, Sachin Puri, Tarsem Moudgil, Carlo Bifulco, Bernard Fox

**Affiliations:** 1 Definiens, Inc., Carlsbad, CA, USA; 2 Definiens AG, Munich, Germany; 3 Earle A. Chiles Research Institute, Providence Cancer Center, Portland, USA

## Introduction

Melanoma is the most serious form of skin cancer, resulting in nearly 10,000 deaths per year in the US alone. Approximately 76,000 people are diagnosed with melanoma each year, and that number has been growing steadily over the last 30 years. Tragically, melanoma affects younger as well as older people. In fact, it is one of the most common forms of cancer in people younger than 30 [1]. A significant challenge impeding melanoma research is that the very cells that cause the cancer, the melanocytes, can confound measurements made using traditional immunohistochemical (IHC) techniques. This is because the brown melanin in the cells creates a high background for bright-field IHC stains, which are often brown or red.

## Results

**Figure 1:**
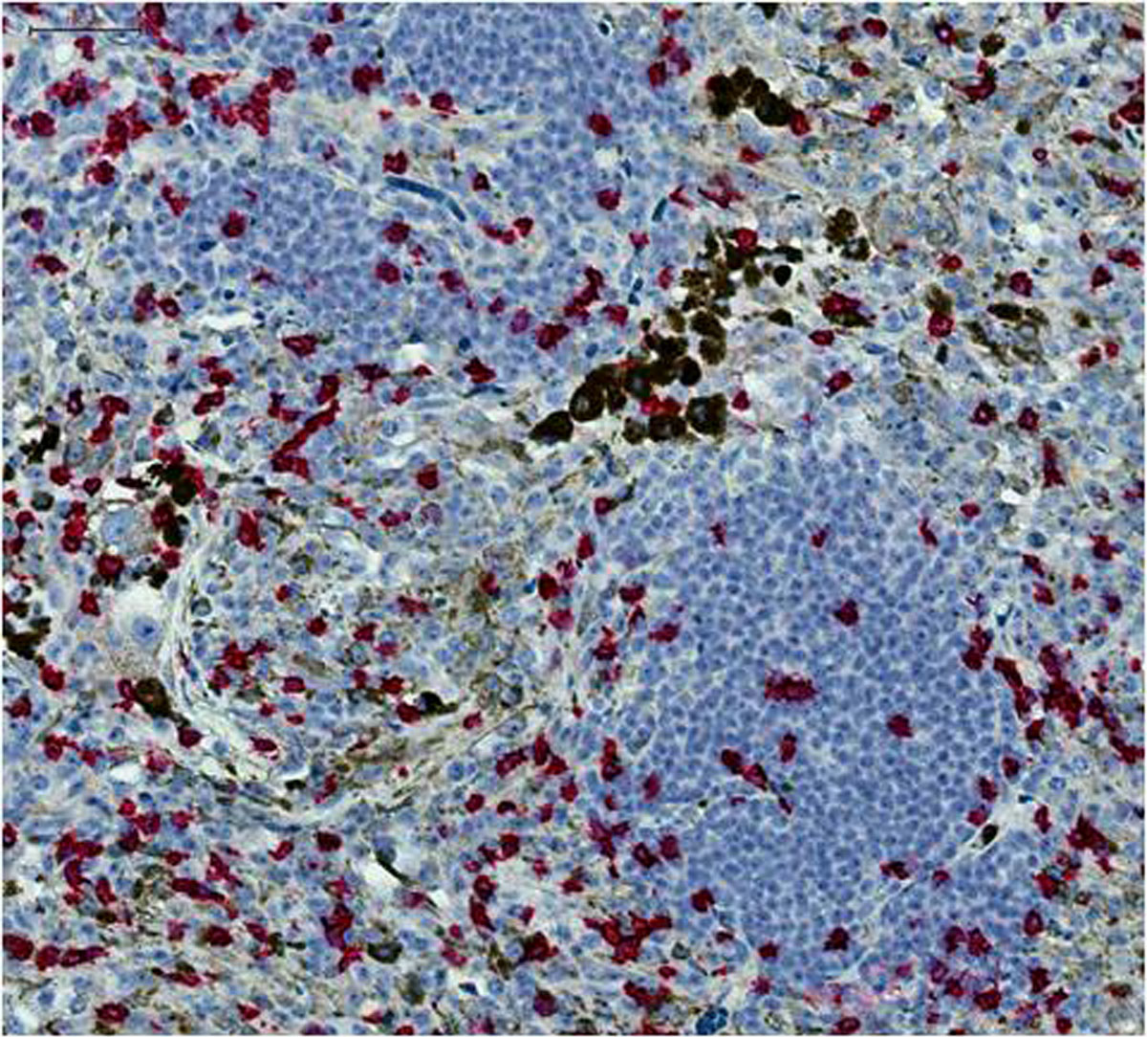
**Original high resolution RGB scan of a classically stained TMA core**.

**Figure 2:**
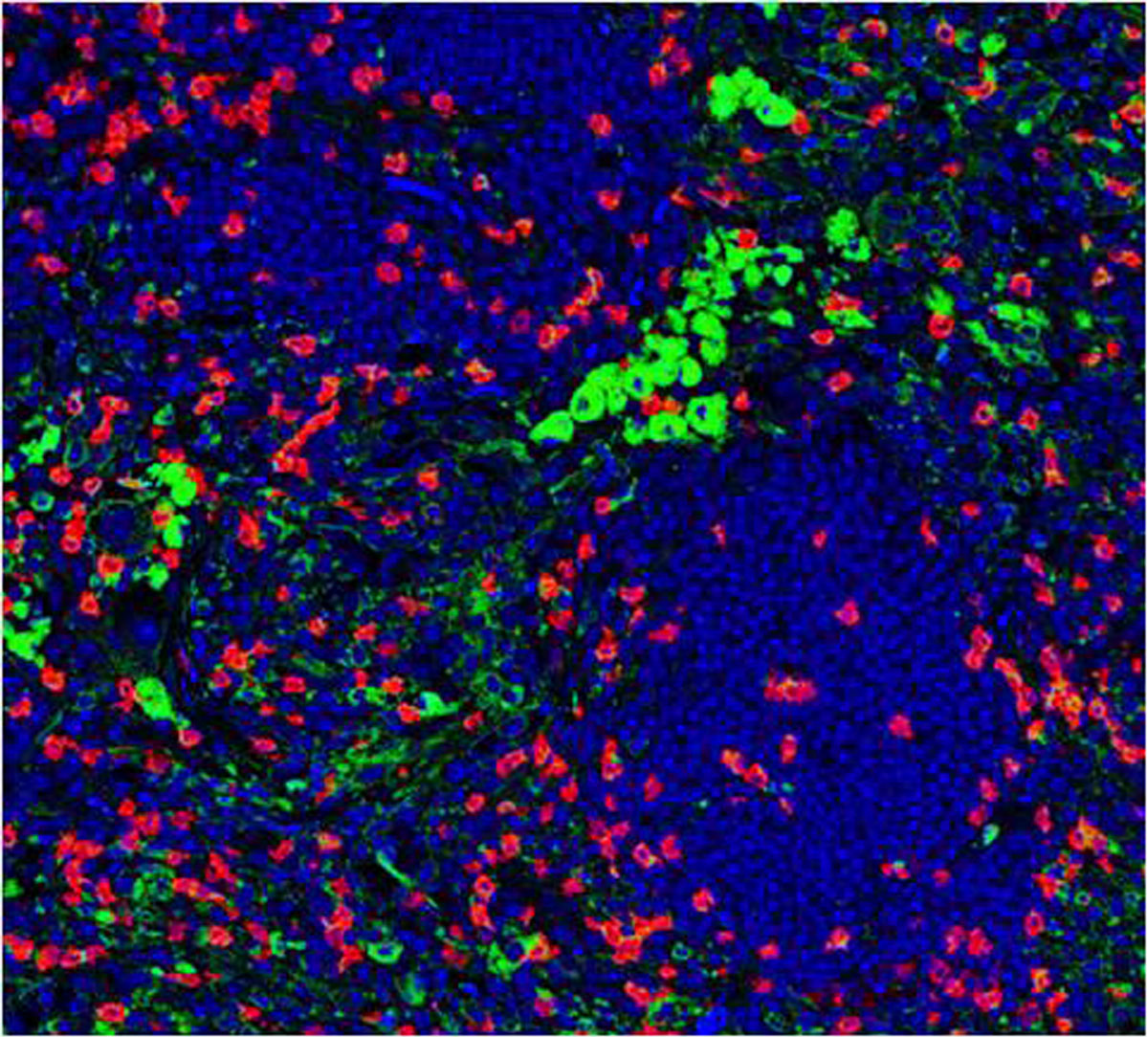
**Pseudo-fluorescent image resulting from the stain isolation algorithm using H**.

A digital bright-field image is made up of separate channels (red, green and blue), with individual intensities determined by a three color CCD acquisition camera. Different colors perceived by eye are a mixture of these three channels, and thus not directly separated by traditional image analysis software. For example, the dark brown melanin filled regions of the image will appear in all three of the channels with different intensities. Definiens' stain isolation algorithm makes use of the Hue-Saturation-Density (HSD) transformation, which is an adaptation of the Hue-Saturation-Intensity model where the optical density for each individual channel is used as opposed to intensity values [2]. We used this powerful technique to successfully separate the red stained CD3 and CD8 positive immune cells from a background of brown melanocytes in TMA core samples from melanoma patients. Each cell type was isolated into a separate channel on the basis of hue. The resulting image is functionally like a fluorescent image, with monochromatic grey scale image for each color (Figure 1 and 2). This greatly simplified and increased the accuracy of the automated detection and quantification of the distinct cell types. We demonstrated rapid and accurate quantification of the immune response that would be virtually impossible using manual techniques.
